# ERK2 phosphorylation of EBNA1 serine 383 residue is important for EBNA1-dependent transactivation

**DOI:** 10.18632/oncotarget.8177

**Published:** 2016-03-18

**Authors:** Ka-Won Noh, Jihyun Park, Eun Hye Joo, Eun Kyung Lee, Eun Young Choi, Myung-Soo Kang

**Affiliations:** ^1^ Department of Health Sciences and Technology, Samsung Advanced Institute for Health Sciences and Technology (SAIHST), Sungkyunkwan University, Seoul, Korea; ^2^ Samsung Biomedical Research Institute (SBRI), Samsung Medical Center and Sungkyunkwan University, Seoul, Korea; ^3^ BioMembrane Plasticity Research Center (MPRC), Seoul National University College of Medicine, Jongno-gu, Seoul, Korea

**Keywords:** Epstein-Barr virus, EBNA1, ERK, phosphorylation, inhibitor

## Abstract

Functional inhibition of Epstein-Barr virus (EBV)-encoded nuclear antigen 1 (EBNA1) can cause the death of EBV infected cells. In this study, a bioinformatics tool predicted the existence of putative extracellular signal-regulated kinase (ERK) docking and substrate consensus sites on EBNA1, suggesting that ERK2 could bind to and phosphorylate EBNA1. In accordance, ERK2 was found to phosphorylate EBNA1 serine 383 in a reaction suppressed by H20 (a structural congener of the ERK inhibitor), U0126 (an inhibitor of MEK kinase), and mutations at substrate (S383A) or putative ERK docking sites. Wild-type (S383) and phosphomimetic (S383D) EBNA1 demonstrated comparable transactivation function, which was suppressed by H20 or U0126. In contrast, non-phosphorylated EBNA1 mutants displayed significantly impaired transactivation activity. ERK2 knock-down by siRNA, or treatment with U0126 or H20 repressed EBNA1-dependent transactivation. Collectively, these data indicate that blocking ERK2-directed phosphorylation can suppress EBNA1-transactivation function in latent EBV-infected cells, validating ERK2 as a drug target for EBV-associated disorders.

## INTRODUCTION

Epstein-Barr virus (EBV) usually infects and replicates in oropharyngeal epithelial cells [1-3]. In latent infections, EBV genome integration is unusual; instead, EBV persists as a multi-copy episome causing lymphomas and carcinomas [4-7] [8-12]. EBV-encoded nuclear antigen 1 (EBNA1) is essential for EBV episome persistence and transcription in dividing cells [13-16]. For this, EBNA1 contains three essential domains: arginine-glycine-rich domain 1 (RG1) (a.a. 61-83), RG2 (a.a. 325-376), and dimerization/cognate DNA binding domain (DD/DBD) (a.a. 459-607) [17, 18]. Both RG1 and RG2 are necessary and sufficient for EBNA1 to associate with its target DNA, and are essential for EBNA1-dependent transcription of latent genes and genome persistence [17, 19-22]. DD/DBD binds specifically to 24 cognate sites in an EBV repetitive sequence that serves as an origin of (latent viral genome) plasmid replication (OriP) and transcription enhancer. DD/DBD enables latent EBV episomes to replicate, while RG domains tether replicated episomes to chromosomes during partitioning. Thus, an EBNA1 inhibitor can potentially terminate latent EBV infection and cancel any effect of EBV in non-malignant and malignant diseases.

Our previous studies identified three selective EBNA1 inhibitors: Roscovitine (a cyclin-dependent kinase1/2 (CDK1/2) inhibitor), H20 (a structural congener of extracellular signal-regulated kinase (ERK) inhibitor), and U0126 (an inhibitor of Mitogen-activated protein kinase kinase 1(MEK1)) [23-25]. While Roscovitine targets multiple CDKs, cellular targets of H20 have been elusive. Surface plasmon resonance showed binding of H20 to a.a. 459-607 of viral EBNA1, and *in silico* analyses suggested that H20 docked in pocket 2 of EBNA1, surrounded by K514, Y518, R521, and P535. Interestingly, this pocket overlapped with the predicted ERK docking motif and the structure of H20 was similar to that of the ERK docking inhibitor (ERKi), which prevented ERK association with the substrate [24]. These findings suggest that H20 could affect ERK2-directed phosphorylation. Here, we provide experimental evidence supporting the role of ERK2 in EBNA1-mediated persistent EBV infection, and propose ERK2 as a novel drug target.

## RESULTS

### Potential ERK2-directed phosphorylation site on EBNA1

**Figure 1:**
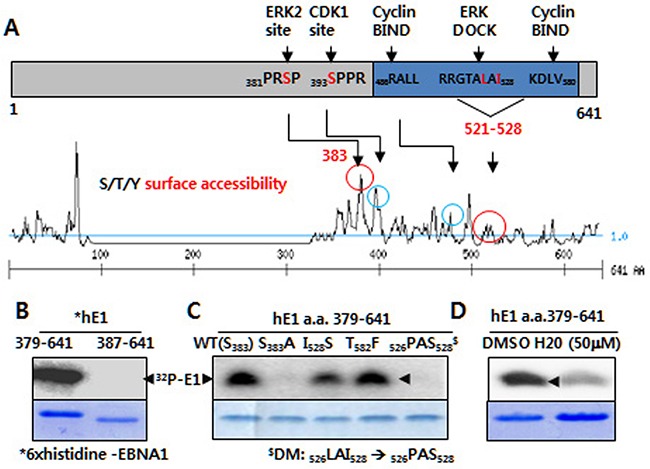
ERK2-directed phosphorylation of EBNA1 S383 *in vitro* **A.** Putative ERK and CDK1 phosphorylation and bindings sites (circled) on EBNA1, as predicted by kinase surface accessibility plots. **B.** *In vitro* kinase assay reveals ERK2 catalytic activity in the presence of 6× histidine-tagged EBNA1 (hE1) a.a. 379-641, but not hE1 a. a. 387-641. Coomassie blue staining shows EBNA1 levels. **C.** Absence of ERK2-directed phosphorylation on hE1 S383A and _526_PAS_528_ double mutant (DM), reduced phosphorylation on I528S mutant. **D.** H20 inhibits ERK-mediated EBNA1 phosphorylation.

Having identified H20 and U0126 as inhibitors of EBNA1, we hypothesized that ERK phosphorylated EBNA1 [23]. We used the ELM, NetPhopho and Motif Scan proteomic tools listed in the Expasy repository (http://www.expasy.org/proteomics), and applied the option of scanning the full length EBNA1 FASTA file (>gi|23893623|emb|CAD53427.1| EBNA-1 protein [Human herpesvirus 4]). The eukaryotic linear motif (http://elm.eu.org) and Netphos 2.0 (http://www.cbs.dtu.dk/services/NetPhos/) tool predicted the existence of a putative motif for ERK2-directed proline-dependent phosphorylation at a.a. 381-384 (PR**S**P), and a potential ERK docking site at a.a. 521-528RRGTALAI) on the surface of EBNA1 [26] (Figure 1A). The Motif Scan tool (http://myhits.isb-sib.ch/cgi-bin/motif_scan) predicted phosphorylation site and surface accessibility. In support of this hypothesis, our previous studies indicated that inhibitors of 18 different kinases redundantly or selectively inhibited EBNA1-dependent transactivation activity [23, 24].

### ERK2, a MAP kinase p42 isoform, phosphorylates S383 on EBNA1 *in vitro*

Using an *in vitro* kinase assay, we observed thatcatalytically active recombinant ERK2 phosphorylated EBNA1 at a.a. 379-641, and 380-394, but not at a.a. 387-641 or 384-398 (Figure 1B; data not shown). Phosphorylation was entirely abrogated in the case of a point mutation at a putative phosphorylation site (denoted S383A), suggesting that ERK2 phosphorylated EBNA1 at serine 383 (S383). In addition, phosphorylation was completely absent in double mutations (DM) of the ERK2 docking motif at a.a. 526-528 _526_LAI_528_ → _526_PAS_528_), but not of the near cyclin-binding motif (T582F). Point mutation at I528S also impaired phosphorylation, albeit less effectively. In accordance with the above data, ERK2-directed phosphorylation of EBNA1 was partially impaired by H20 [24]. Given that the *in* vitro kinase assay lacked MEK1, U0126 had no effect on ERK2-directed EBNA1 phosphorylation (data not shown). Moreover, the *in vitro* kinase assay clearly showed that the ERK docking motif was required for ERK2-directed phosphorylation at EBNA1 S383.

### ERK2 associates with EBNA1 *in vivo*

**Figure 2:**
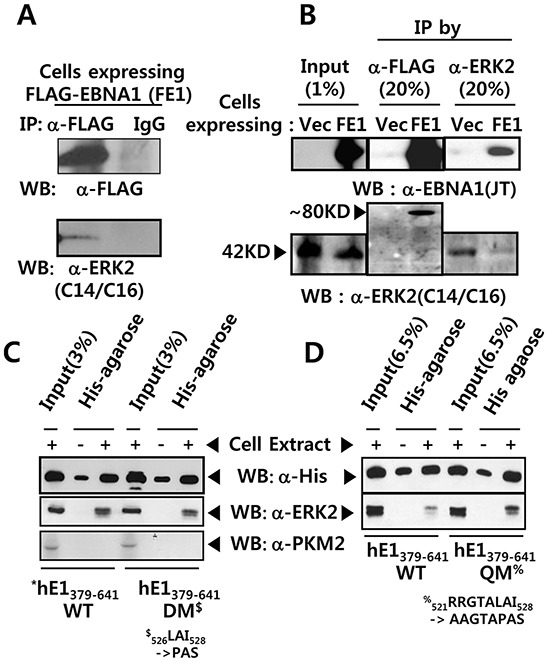
EBNA1 forms a complex with ERK2 **A.** Anti FLAG M2 antibody pulled-down FLAG-tagged EBNA1 (FE1) and ERK2 in BJAB cell extract, while IgG control antibody did not. **B.** Reciprocal co-immunoprecipitation (co-IP) of FE1 and cellular ERK2. Stable BJAB cells expressing vector control (Vec) or FE1 were immunoprecipitated using anti-FLAG and anti-ERK2 antibodies. 1% input and 20% immune complex were resolved on 10% SDS protein gels and probed with anti-FLAG, anti-EBNA1, and anti-ERK2 antibodies. Despite low ERK2 IP efficiency in FE1 cells, substantial FE1 was co-immunoprecipitated by anti-ERK2 antibody. The low ERK2 IP efficiency in FE1 cells, compared to vector control, was due to experimental deviation seen in this specific case (See also Supplementary Figure S1). **C–D.** Pull-down assay using His-bind magnetic beads, followed by western blotting using anti-His tag and anti-ERK2 antibodies. Interaction with ERK2 was observed between hE1 a.a. 379-641 WT, DM, and quadruple mutant (QM) (_521_RRGTALAI_528_ → _521_AAGTAPAS_528_). To rule out hE1 non-specific interaction with other cellular kinases, the same membrane was stripped and reprobed with anti-PKM2 antibody. Results show that mutations in the putative ERK docking motif of EBNA1 did not abrogate interaction between EBNA1 and ERK2.

The presence of an ERK docking motif on EBNA1 led us to test whether ERK associated with EBNA1 via this motif. Using an anti-FLAG (M2) antibody, we were able to pull down FLAG-tagged EBNA1 (FE1) and, less efficiently, ERK2 (Figure 2A), but not the isotype control. This was further confirmed by reciprocal immunoprecipitation (IPs), whereby anti-ERK2 efficiently pulled down EBNA1 (Figure 2B; Supplementary Figure S1). Contrary to the hypothesis, DM in the ERK docking motif did not abrogate the interaction between EBNA1 and ERK2 (Figure 2C). To our surprise, a quadruple mutation (QM) within a.a. 521-528 (_521_RRGTALAI_528_ → _521_AAGTAPAS_528_) further increased the interaction (Figure 2D). This indicates the presence of redundant ERK docking motif sites on EBNA1 between a.a. 379 and 641. Taking together, these results suggest that ERK2 associates with EBNA1 directly via EBNA1 a.a. 521-528 (Figure 1C), and indirectly via an unknown protein linker between a.a. 379-641 (Figure 2C, 2D).

### ERK2-directed phosphorylation of EBNA1 contributes to EBNA1-dependent transactivation

**Figure 3:**
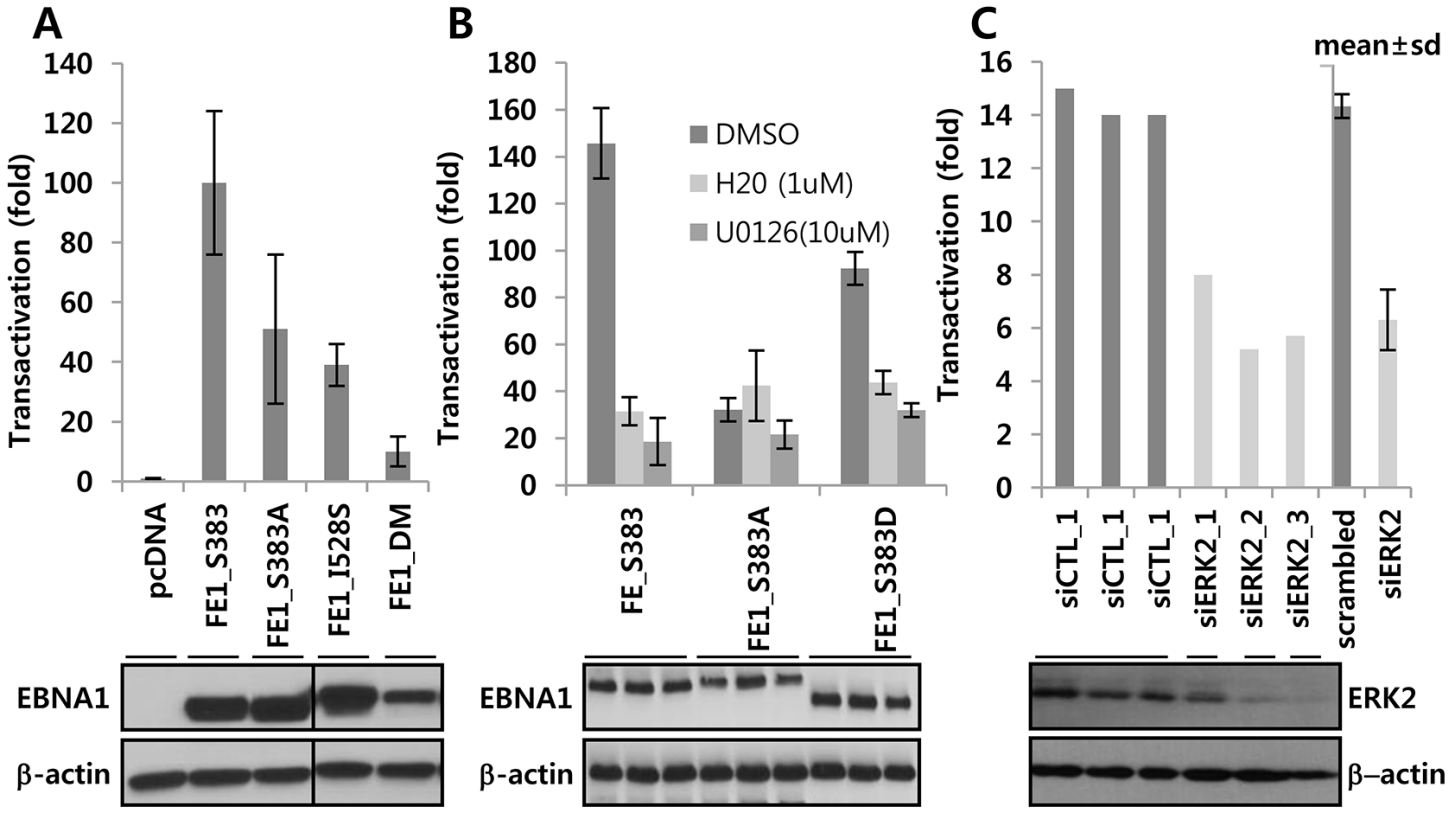
ERK2-directed phosphorylation contributes EBNA1-dependent transcriptional activity **A.** EBNA1-negative BJAB cells were transiently co-transfected with a control vector, EBNA1 S383, S383A, S528, and PAS, along with EBNA1-dependent (POLP2) and -independent (SV40p-RL) reporters. **B.** After transient co-transfection of indicated expression plasmids with reporters (see above), cells were divided into three aliquots, and treated with ERKi in the absence or presence of DMSO, H20 (0.5 μM), and U0126 (10 μM). Slight difference in EBNA1 protein size resulted from variations in glycine-alanine (GA) repeats during PCR, as can be seen from Western blots underneath. **C.** Use of scramble siRNA or three different siRNAs against ERK2 (siERK2_1, _2, _3) resulted in partial knockdown 3 days post transfection of BJ-FE1 cells. These and control (siCTL) cells were transiently transfected with POLP2 and SV40p-RL reporters and assayed after 2 days. Note that the lower transactivation (~15 fold) is due to use of a stable BJAB cell line constitutively expressing FE1, which is usually lower than transiently transfected FE1.

Wild-type (WT) EBNA1 (S383), phosphorylation-deficient alanine (S383A), and phosphomimetic peptide (S383D) substitutions were tested for transactivation function in a full-length FE1 background in the presence or absence of U0126, and H20, or ERK2 siRNA. While transcriptional activity of WT EBNA1 was >140 fold, S383A and S383D had only 32 fold (22% of WT) and 92 fold activity (~66% of WT), respectively (Figure 3A). WT- and S383D-dependent transactivation functions were repressed by H20 [24] and U0126. This additional inhibitory effect was significantly diminished in S383A, further confirming that S383 was the primary target for U0126 and H20 (Figure 3B). In another assay based on EBNA1-dependent transactivation of an OriP-harboring reporter, EBNA1 S383A, I528S, or DM showed much lower activity (< ~50%, 39%, and 10%, respectively) (Figure 3A). Next, we tested the effect of siRNA-mediated depletion of ERK2 on EBNA1-dependent transactivation. Unlike ERK1 siRNA, ERK2 siRNA could deplete its target transcript by >50 % within 3 days after transient transfection. In multiple independent experiments using BJAB Burkitt's lymphoma cells, ERK2 knockdown decreased transcription of the the EBNA1-dependent reporter but not of the co-transfected EBNA1-independent SV40p-Renilla luciferase (SV40p-RL) reporter (Figure 3C).

**Figure 4:**
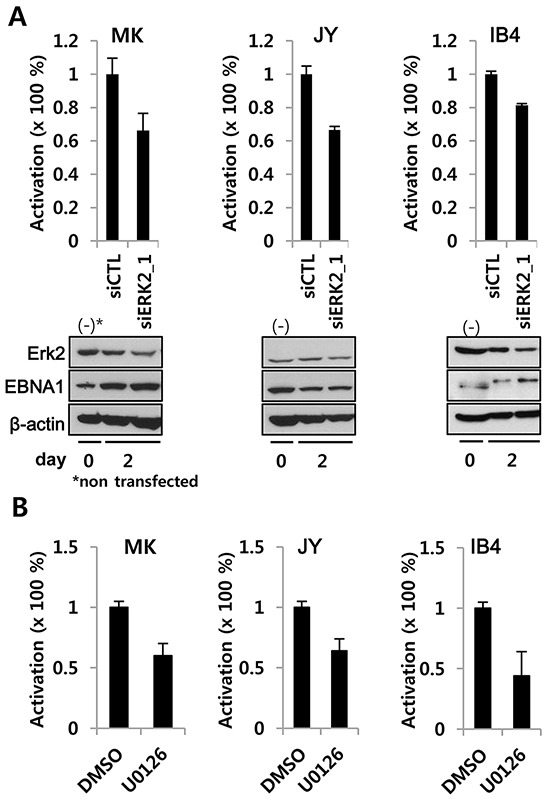
ERK2 depletion or inhibition decreases EBNA1-dependent transactivation activity in EBV-transformed LCL **A.** siRNA-mediated transient knockdown of ERK2 in LCL reduced EBNA1-dependent transactivation. EBV-immortalized fresh LCLs (MK, JY) and an old LCL (IB4) were transiently co-transfected with POLP2, EBNA1-independent SV40p-RL along with siCTL (scrambles control siRNA) or siRNA2_1(100 nM) (See Figure 3). **B.** LCLs transiently transfected with POLP2 and SV40p-RL were divided in two aliquots, treated for 2 days with U0126 (10 μM) or DMSO, and subjected to reporter assay. In A and B, EBNA1-specific firefly luciferase was normalized to EBNA1-independent RL. SiERK2 and U0126 activities were compared to the normalized activity in siCTL or DMSO treated cells, where it was set to 100%.

To better understand the above findings in the context of EBV biology, we performed the reporter assays in two freshly EBV-immortalized lymphoblastoid cell lines (LCLs), MK and JY, and an old LCL, IB4. ERK2 siRNA specifically reduced EBNA1-mediated OriP-linked firefly luciferase reporter activity (normalized to co-transfected SV40p-RL), while scramble siRNA-treated cells were indistinguishable from negative controls. It should be noted that ERK2 knockdown was less robust in LCL (Figure 4) than in BJAB cells (Figure 3), possibly due to lower transfection efficiency [27]. In addition, treatment of LCLs with U0126 lowered EBNA1-dependent transactivation after only 2 days (Figure 4B).

### Treatment with U0126 or S383A mutation have no significant effect on EBNA1 nuclear or cytoplasmic localization

Serine 383 is located within the EBNA1 nuclear localization signal (a.a. 379-386), where several serines control EBNA1 localization [28]. To explore the effect of mutation or U0126 treatment on EBNA1 sub cellular localization, BJAB cells stably expressing FLAG-EBNA1 S383 (FE1 S383) or S383A (FE1 S383A) were treated with dimethyl sulfoxide (DMSO) (-) or U0126 (10 μM) for 4 days. Nuclear and cytoplasmic fractions were isolated and probed with antibodies against EBNA1, transcription factor Sp1 (nucleus), α-tubulin (cytoplasm), and β-actin (normalization). No substantial differences in EBNA1 subcellular localization or expression could be found between WT and S383A EBNA1.expressing cells upon U0126 treatment (Supplementary Figure S2A). Similarly, live cell confocal microscopy showed that the subcellular localizations of stably expressed EGFP-EBNA1 S383 or S383A were almost identical in BJAB cells. This indicates that the S383A mutation did not affect EBNA1 subcellular localization or shuttling between nucleus and cytoplasm (Supplementary Figure S2B).

## DISCUSSION

The bioinformatics ELM tool predicted that 25 serines on EBNA1 could be redundantly phosphorylated by at least 10 different serine/threonine kinases. These include: protein kinase A, casein kinase 1 (CK1), casein kinase 2 (CK2), glycogen synthase kinase 3 (GSK3), never in mitosis A-related kinases 2, phosphoinositide-3-OH-kinase related kinases (a.a. 383-389), protein kinase B, polo-like kinase, proline-directed kinases (such as MAPK or ERK) (a.a. 380-386, 390-396), calcium/calmodulin-dependent protein kinase type II (CaMKII), and CDK1. EBNA1 can be phosphorylated on multiple serines, though not on threonines or tyrosines [29, 30]. It is experimentally proven that CK2, CDKs, and the only EBV kinase analogue to cellular CDKs (BGLF4) can phosphorylate multiple serine residues on EBNA1 [31] [23] [32, 33].

**Figure 5:**
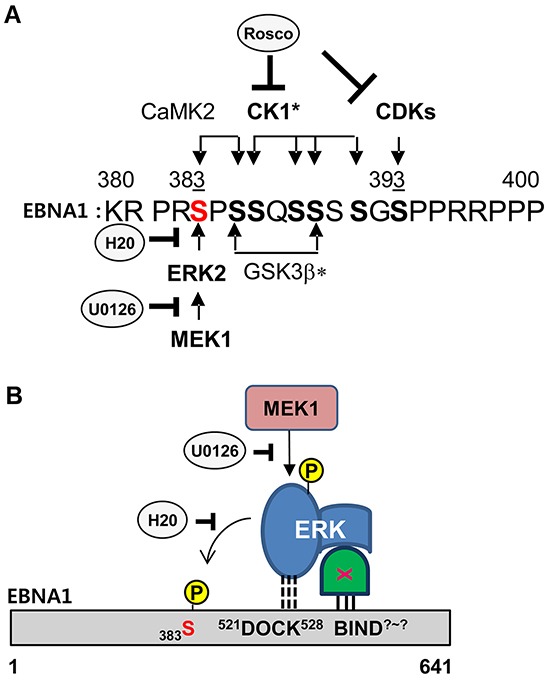
Model of ERK2-EBNA1 interaction **A.** Schematic diagram of putative phosphorylation cascades in EBNA1 a.a. 380-400. Consensus sites and comments for indicated kinase are as follows: ERK2 (Px**S/T**P or xx**S/T**P, CaMK II (Rxx**S/T**), CDK1 (**S/T**PxR/K), CK1* (Sxx**S/T**), GSK3b* (**S**xxxS), bold S/T (phosphorylation site), underlined S (prior phosphorylation site needed for subsequent phosphorylation at bold S), *cascade (prior phosphorylation)-dependent phosphorylation. **B.** Model of EBNA1-ERK2 interaction. A putative ERK docking motif (^521^DOCK^528^) on EBNA1 mediates a direct interaction with ERK2 and phosphorylation at S383 but is dispensable for *in vivo* interaction. An unknown protein linker (X) likely mediates ERK2 and EBNA1 interaction *in vivo* through an unknown site (BIND^?-?^) on EBNA1.

Taken together, we present a schematic diagram of the kinase cascade and ERK2-EBNA1 interaction (Figure 5). S393 is phosphorylated by CDK/cyclin complexes, and the S393A mutation was previously described as having no effect on transcription activity and only 60% persistence [23]. In this study, S383 has emerged as the site for ERK2 phosphorylation of EBNA1 (Figure 5A). Multiple kinases participate in phosphorylation on a.a. 380-390, and prior phosphorylation is often required for subsequent phosphorylation cascades. For example, ERK2- or CaMKII-mediated phosphorylation at S383 in EBNA1 may enhance CK1-dependent phosphorylation on S386 (Figure 5A). In addition to S393, here we provide evidence of the importance of S383 for EBNA1-dependent transactivation. Although we do not know how ERK2-directed phosphorylation affects EBNA1 activity, phosphorylated S383 may alter EBNA1 protein stability or change its affinity towards interacting proteins. To this end, the S385A mutation has lower binding affinity for the nuclear import adaptor protein, Importin subunit alpha 5 [28].

The *in vitro* kinase assay showed a potential direct interaction between EBNA1 a.a.379-641 and ERK2 (Figure 5B). DM abolished ERK2-mediated phosphorylation *in vitro*, indicating that the original sequence was required for association-dependent phosphorylation. Given that DM could not abolish this interaction *in vivo*, and QM even augmented it, we speculate there is a secondary site in EBNA1 responsible for direct or indirect association with ERK2 (Figure 5B). Bioinformatics analysis did not predict any direct ERK docking sites on EBNA1 other than a.a. 521-528. This leaves the possibility of a protein linker regulating the indirect association of ERK2 with EBNA1 a.a. 379-641. The identity and mechanistic role of the protein linker and docking site are beyond the scope of this study. It is nevertheless clear that S383 and the ERK docking motif are required for ERK2-directed phosphorylation of EBNA1 and its EBNA1 transactivation function. Therefore, it is equally possible that other kinases, such as viral BGLF4, CDKs, or CKs, may phosphorylate S383A *in vivo* [31] [23] [32].

Finally, this study provides a rationale for why epidermal growth factor receptor (EGFR) inhibitors were found to block also EBNA1 [23]. Given that many EGFR signaling pathways converge on ERK2, EGFR inhibitors negatively affect ERK2 activity, and thus actively repress EBNA1-dependent transactivation [23].

## MATERIALS AND METHODS

### Plasmids and chemicals

FLAG-EBNA1 (FE1), enhanced green fluorescent protein-tagged EBNA1 (EGFP-EBNA1), and EBNA1-dependent reporters (OriP-EBV), C promoter-driven firefly luciferase (OriPCp-FL) and OriP-enhancer linked minimal promoter-driven firefly luciferase with puromyin cassette (POLP2) have been described previously [20] [23]. EBNA1-independent reporters, pSV40 promoter-driven *Renilla* luciferase (RL) or pGK promoter-β galactosidase (pGK- βgal), were used to estimate cell viability. Six histidine-tagged EBNA1 sequences (hE1) (a.a. 387-641, 379-641, 459-607) were cloned into plasmid pET28a (Invitrogen Life Sciences, Carlsbad, CA) [23]. EBNA1 mutants S383A, PAS (double mutation; DM), AAGTAPAS (quadruple mutation; QM) were generated using Quick Change (Stratagene, La Jolla, CA). Primers are listed in Supplementary Table S1. FE1_S383 (131 glycine-alanine repeats), S383A (131 GA), S383D (<123 GA), I528S (~133GA), DM (~133 GA), and QM (131 GA) were generated using a full length FE1 WT S383 template with all 235 GA repeats (a.a. 90-324) [20] [23]. Primers are listed in Supplementary Table S1. Consistent with previous reports the length of GA repeats did not affect EBNA1 transactivation activity [34]. In addition, EGFP-EBNA1 S383A was made using EGFP-EBNA1 as template [20]. Partial or complete GA removal, resulting from an in-frame deletion during PCR, did not affect EBNA1 functionality unless there were fewer than 25 GA repeats [20, 35, 36]. U0126 was purchased from Cell Signaling Technology (catalog no. 9903; Danvers, MA) and H20 was synthesized as previously described by the authors [24].

### Cell lines and reporter assay

EBV-negative human Burkitt's lymphoma cells (BJAB), BJAB cells that stably expressed FE1 (BJ-FE1), BJ-FE1 cells with EBNA1-dependent episomal OriPCp-FL (or POLP2), and BJ-FE1 with integrated EBNA1-independent SV40p-RL (BJ-FE1-OF) have been described previously [20]. Where necessary, EBV-immortalized fresh LCLs (MK, JY) were established from peripheral blood as described [27]. For the EBNA1-transactivation reporter assay, cells were transiently co-transfected with EBNA1-dependent and independent reporters (and EBNA1 in case of BJAB cells), and then aliquoted for treatment with the indicated compounds. Cell extracts were subjected to reporter assays 2-3 days after transfection [23] using Dual-Glo Luciferase reporter system (Promega, Madison, WI). When necessary, ERK2 transcripts were depleted in BJ-FE1 cells for 2-4 days by transfecting siRNAs specific for ERK 1, ERK2, or a scramble sequence (Supplementary Table S1). Next, EBNA1-dependent POLP2 and EBNA1-independent SV40p-RL reporters were transiently transfected to assess the effect of ERK depletion on EBNA1-dependent transactivation. In LCLs, cells were transiently transfected with PLOP2 and SV40p-RL, divided into two, and treated for 2 days with U0126 (10 μM) or DMSO. EBNA1-specific firefly luciferase was normalized to EBNA1-independent RL. siERK2 or U0126 activity were compared to the normalized activity of control siRNA (siCTL) or DMSO-treated cells, which was set to 100%.

### Subcellular localization of EBNA1

Cytoplasmic and nuclear proteins from BJ-FE1 S383 WT, and S383A MT cells treated with DMSO and U0126 for 3 days were washed in 1 mL of ice cold PBS and then fractionated. Cytoplasmic extracts were obtained using 5× hypotonic buffer (10 mM HEPES pH 7.9, 10 mM KCL, 0.1 mM EDTA, 0.1% Triton X-100, 1× protease inhibitor cocktail); nuclear extracts were obtained using 1× hypertonic buffer (20 mM HEPES pH 7.9, 0.4 M NaCl, 1 mM EDTA, 25% glycerol, 1× protease inhibitor cocktail). Extracts were stored at −80°C until assayed. Subcellular localization of EGFP-EBNA1 S383 WT and S383A MT in BJAB cells was assayed by live confocal microscopy as described previously [20].

### Purification and kinase assay of 6× histidine-EBNA1

Different EBNA1 constructs (387-641 WT, 379-~641 WT, or MTs of S383A, I528S, T582F, PAS (DM), and AAGTAPAS (QM)) were tagged with 6× histidine at the N-terminus and purified from *Escherichia coli* BL21 (DE3) Rosetta/pLysS cells as described previously [23]. Each purified EBNA1 was incubated with 20 U catalytically active recombinant ERK2 (NEB, Beverly, MA), in the supplied buffer containing 200 μM ATP and [γ^32^P] ATP in the presence or absence of compounds, as indicated previously [23].

### Immunoprecipitation

Cell extracts from stable BJAB cells expressing vector control or FE1 were prepared inlysis buffer (50 mM Tris-HCL, pH 7.4, 150 mM NaCl, 1.5 mM EDTA, 1% NP40, 3% glycerol, 0.5 μg/ml leupeptin, 0.1 μg/ml aprotinin). Cell extracts were precleared with Protein G/A agarose beads (10% v/v of 50% bead slurry), washed 3× with 1% lysis buffer, and immunoprecipitated using anti-FLAG, anti-ERK2, or IgG isotype control antibodies as described previously [23]. Immune complexes were resolved on 10% sodium dodecyl sulfate (SDS) protein gels, blotted and probed with anti-FLAG, anti-EBNA1 (JT anti-EBV human serum, or EBNA1 monoclonal antibody from Advanced Biotechnology Inc., Eldersburg, MD), and anti-ERK2 (C14/C16; Santa Cruz, Dallas, TX) antibodies. Alternatively, 6× histidine-tagged EBNA1 a.a. 379-641 WT, DM, and QM were incubated each with either buffer control or BJAB cell extracts (10^6^ cells) as a source of cellular kinases (i.e., ERK2), subsequently pulled-down by His-bind magnetic agarose beads (EBE-1038; Elpis Biotech, Daejeon, Korea), washed and subjected to western blotting using anti-His-tag, anti-ERKs, and anti-pyruvate kinase muscle type isoform 2 (PKM2) antibodies.

## SUPPLEMENTARY INFORMATION


