# Supplementary material for: ERK2 phosphorylation of EBNA1 serine 383 residue is important for EBNA1-dependent transactivation
Source: Oncotarget. 2016 Mar 18;7(18):25507–15. doi: 10.18632/oncotarget.8177 (PMC5041921; doi:10.18632/oncotarget.8177)
Supplement: Supplementary file 1 [file oncotarget-07-25507-s001.pdf]

SUPPLEMENTARY INFORMATION

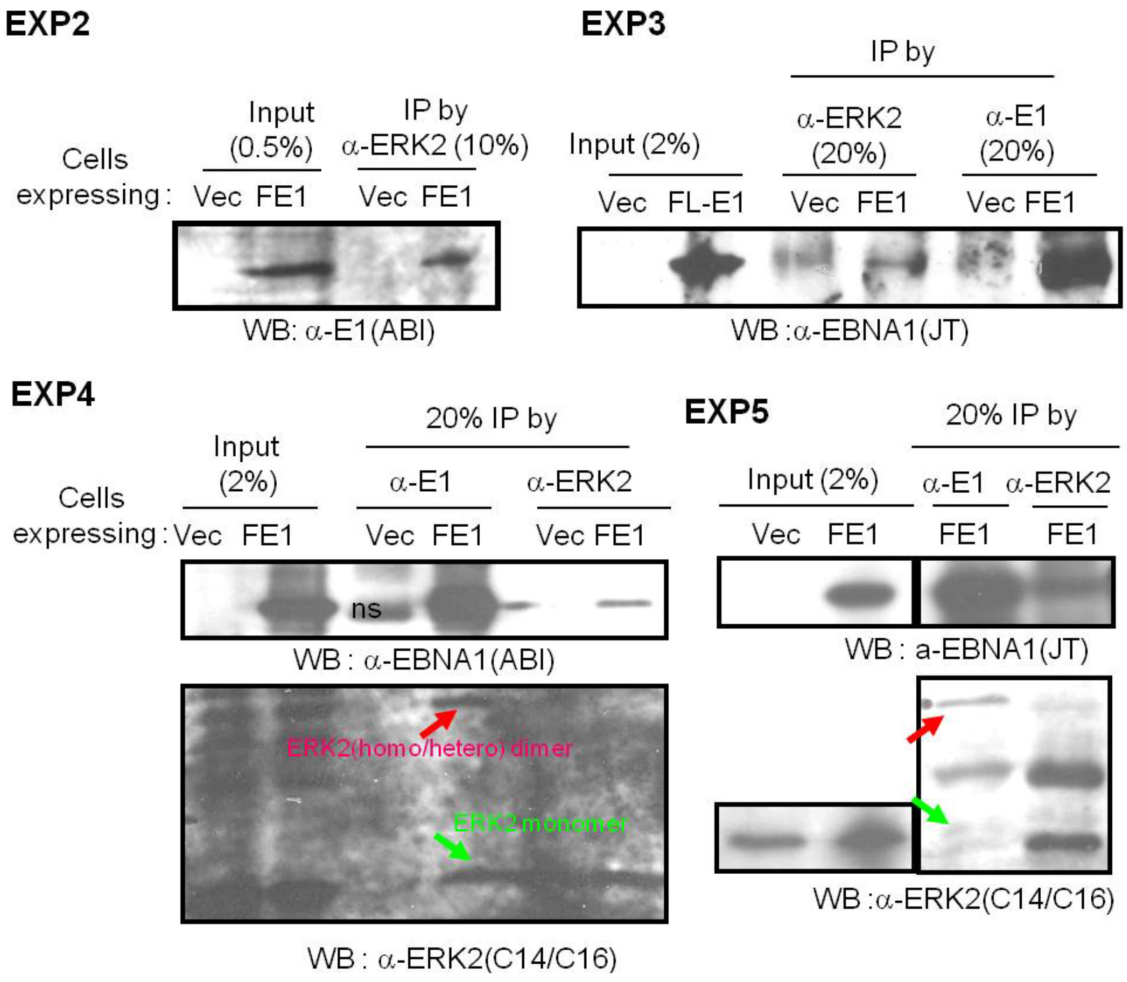

Supplementary Figure S1: Additional data supporting EBNA1-ERK2 interaction *in vivo*. A. (EXP 2) Immune precipitation (IP) by anti-ERK2 antibody of vector- or FLAG-EBNA1 (FE1)-expressing BJAB B cell extracts followed by probing with anti-EBNA1 antibody. (EXP 3). Reciprocal IPs by anti-ERK2 and anti-EBNA1 antibodies of vector- or FE1-expressing BJAB B cell extracts followed by probing with anti-EBNA1 (JT human polyclonal serum). **B.** (EXP 4, 5) Reciprocal IPs and western blots with indicated antibodies. Note that, there were two species of EBNA1-bound ERK2 on SDS-PAGE: a larger ERK2 (likely ERK2 homodimer or heterodimer in complex with EBNA1; red arrow), and a smaller ERK2 of its original size (green arrow). The slower migrating ERK2 likely results from insufficient denaturation before sample loading.

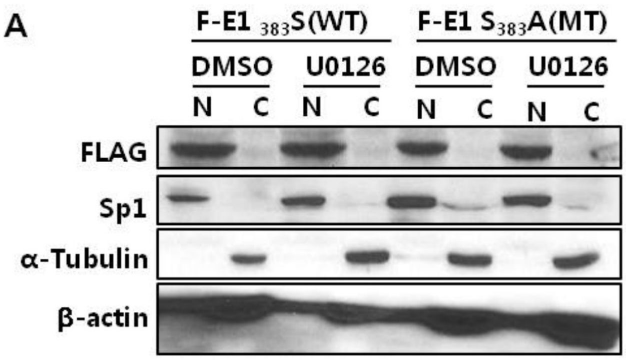

B

	Number and fraction (%) of cells EBNA1 localized in			
EGFP-EBNA1	Nucleus	Cytosol	Nuc/Cyt	Total
WT S_{383}	26	4	5	35
Fraction	74%	11%	14%	100%
MT S_{383}A	16	1	4	21
Fraction	76%	5%	19%	100%

Supplementary Figure S2: Effect of U0126 or S383A mutation on EBNA1 nuclear and cytoplasmic localization. A. BJAB cells stably expressing FLAG-EBNA1 S383 WT (FE1 S383) or S383A MT (FE1 A383) were treated with DMSO (-) or U0126 (10 µM) for 4 days. Nuclear (N) and cytoplasmic (C) fractions were isolated and probed with mouse antibodies against EBNA1, Sp1 (a nuclear protein marker), α-tubulin (cytoplasmic protein marker) and β-actin (a protein loading control). **B.** BJAB cells expressing EGFP-EBNA1 S383 WT or S383A MT in log phase were visualized by confocal microscopy. EBNA1 subcellular localization for at least 21 individual cells was determined. Mutation did not significantly affect EBNA1 nuclear or cytoplasmic localization.

Supplementary Table S1: List of primers used for siRNA construction, site-directed mutagenesis and expression in this study

Usage for	Name	strand	Sequence (5′ to 3′)
siRNA	siERK1-1	Forward	CAUGAGAGAUGUCUACAUUUU
		Reverse	AAUGUAGACAUCUCUCAUGUU
	siERK1-2	Forward	CUGUACAAGUUGCUGAAAAUU
		Reverse	UUUUCAGCAACUUGUACAGUU
	siERK1-3	Forward	GCUUCCUGACGGAGUAUGUUU
		Reverse	ACAUACUCCGUCAGGAAGCUU
	siERK2-1	Forward	GAACAUCAUUGGAAUCAAUUU
		Reverse	AUUGAUUCCAAUGAUGUUCUU
	siERK2-2	Forward	CAAAUGAAAGAUGUAUAUAUU
		Reverse	UAUAUACAUCUUUCAUUUGUU
	siERK2-3	Forward	GCAGAAAUGCUUUCUAACAUU
		Reverse	UGUUAGAAAGCAUUUCUGCUU
	Negative control	Forward	ACGUGACACGUUCGGAGAAUU
		Reverse	UUCUCCGAACGUGUCACGUUU
Site-directed mutagenesis	$S_{383}A$	Forward	GAAAAGAGGCCCAGGGCTCCCAGTAGTCAGTCATC
		Reverse	GATGACTGACTACTGGGAGCCCTGGGCCTCTTTTC
	$S_{383}D$	Forward	GAAAAGAGGCCCAGGGATCCCAGTAGTC
		Reverse	GACTACTGGGATCCCTGGGCCTCTTTTC
	$L_{526}P$	Forward	CGAGGAACTGCCCCTGCTATTCCACAATGTCGTC
		Reverse	GACGACATTGTGGAATAGCAGGGGCAGTTCCTCG
	$_{526}PAS_{528}$(DM)	Forward	CGAGGAACTGCCCTTGCTAGTCCACAATGTCGTC
		Reverse	GACGACATTGTGGACTAGCAAGGGCAGTTCCTCG
	$T_{582}F$	Forward	CGATTAAGGACTTTGTTATGACAAGCCCGC
		Reverse	GCGGGCTTTGTCATAACAAAGTCCTTAATCG
	$*_{521}AAGTAPAS_{528}$ (QM)	Forward	TCCCTTTACAACCTAGCGGCAGGAACTGCC*CCT*GCT
		Reverse	AGCAGGGGCAGTTCCTGCCGCTAGGTTGTAAAGGGA
Expression	E1 a.a. 459-607	forward	CGTCGACATATGCGCAAAAAAGGAGGGTGGTTTGG
	(18a)	reverse	CGTCGAAAGCTTTCAAGGCAAATCTACTCCA TCGTCAAAGC
	E1 a.a. 379-607	forward	CGTCGACATATG AAGAGGCCCAGGAGTCCCA GTAGTCAG
	(42b)	reverse	CGTCGAAAGCTTTCACTCCTGCCCTTCCTCAC CCTCATC
	E1 a.a. 386-641	forward	CGTCGACATATGCAGTCATCATCATCCGGGTCT CCACCG
	(42c)	reverse	CGTCGAAAGCTTTCACTCCTGCCCTTCCTCACCC TCATC

*Template for QM was DM, Mutated sites are underlined
